# Effective lung nodule detection using deep CNN with dual attention mechanisms

**DOI:** 10.1038/s41598-024-51833-x

**Published:** 2024-02-16

**Authors:** Zia UrRehman, Yan Qiang, Long Wang, Yiwei Shi, Qianqian Yang, Saeed Ullah Khattak, Rukhma Aftab, Juanjuan Zhao

**Affiliations:** 1 College of Computer Science and Technology (College of Data Science), Taiyuan University of Technology, Taiyuan, China; 2 School of Software, North University of China, Taiyuan, China; 3 Jinzhong College of Information, Jinzhong, China; 4 NHC Key Laboratory of Pneumoconiosis, Shanxi Key Laboratory of Respiratory Diseases, Department of Pulmonary and Critical Care Medicine, The First Hospital of Shanxi Medical University, Taiyuan, Shanxi China; 5 Centre of Biotechnology and Microbiology, University of Peshawar, Peshawar, 25120 Pakistan

**Keywords:** Computational biology and bioinformatics, Computational models

## Abstract

Novel methods are required to enhance lung cancer detection, which has overtaken other cancer-related causes of death as the major cause of cancer-related mortality. Radiologists have long-standing methods for locating lung nodules in patients with lung cancer, such as computed tomography (CT) scans. Radiologists must manually review a significant amount of CT scan pictures, which makes the process time-consuming and prone to human error. Computer-aided diagnosis (CAD) systems have been created to help radiologists with their evaluations in order to overcome these difficulties. These systems make use of cutting-edge deep learning architectures. These CAD systems are designed to improve lung nodule diagnosis efficiency and accuracy. In this study, a bespoke convolutional neural network (CNN) with a dual attention mechanism was created, which was especially crafted to concentrate on the most important elements in images of lung nodules. The CNN model extracts informative features from the images, while the attention module incorporates both channel attention and spatial attention mechanisms to selectively highlight significant features. After the attention module, global average pooling is applied to summarize the spatial information. To evaluate the performance of the proposed model, extensive experiments were conducted using benchmark dataset of lung nodules. The results of these experiments demonstrated that our model surpasses recent models and achieves state-of-the-art accuracy in lung nodule detection and classification tasks.

## Introduction

Lung cancer is the main reason for cancer-related deaths, according to the American Cancer Society. Following to the statistics for cancer in 2022, there were almost 1.9 million reported cases and a total of 609,360 deaths. Nearly 350 of these deaths each day were caused by lung cancer^1^. Despite medical improvements, cancer continues to be a serious health concern, and it is still very difficult to successfully treat and prevent its many forms. Cancer therapy is complicated and difficult due to its many kinds. Furthermore, certain tumors can be fatal, emphasizing the importance of early detection^2^. Screening is critical for detecting cancer in its early stages since it looks for cancer cells in patients who are asymptomatic. This stage is critical in the battle against cancer because it allows for prompt detection, which is required for effective treatment. Medical imaging systems provide important information about the kind and stage of cancer that may be used to build a suitable treatment strategy^3,4^. As a result, it is critical to offer clinical follow-up for patients and to undertake cancer tests in order to detect cancer early. This method facilitates in treatment planning and, as a result, improves patient outcomes^5^.

The quality of the data collected utilizing scanning technologies has a considerable impact on the accuracy of sickness diagnosis and treatment findings. Precise analysis based on reliable screening processes and treatment regimens can improve patients’ overall quality of life and length of life. The use of modern cancer imaging technology is required to reveal the incredibly effective treatment regimens. Patients who undergo the necessary imaging tests and inspection have a significant advantage over other patients during the arduous treatment process. A comprehensive analysis of imaging data is incredibly important in order to obtain the finest treatment plans and, ultimately, improve patient outcomes.

The expenses of screening procedures for lung nodules are considerable, and it can be difficult to recognize abnormalities since nodules come in a diversity of sizes and forms. In order to tackle this challenging endeavor, computer-aided diagnostic (CAD) systems have emerged as crucial tools for physicians. Positive results from recent research on machine learning-based digital pathology picture categorization point to the possibility of a rise in the use of these systems in pathology clinics. The use of AI and machine learning-based solutions is expected to significantly increase in the future, particularly within the discipline of pathology.

The most lethal type of cancer is lung cancer, however early identification can significantly improve the prognosis for patients. Low-dose computed tomography has become the gold standard for identifying which lung nodules need a biopsy to evaluate if they are malignant or benign. In clinical settings, this approach has a comparatively high risk of false positives. It frequently requires the identification of a sizable number of possibly cancerous nodules for biopsy, resulting in a great deal of unneeded biopsies being carried out on people who aren’t genuinely suffering from cancer.

1. We developed a custom CNN architecture with integrated channel and spatial attention mechanisms enhances feature extraction by selectively focusing on relevant features, improving accuracy in image classification.
2. The inclusion of attention mechanisms addresses limitations of traditional CNNs, allowing the model to emphasize important patterns and suppress noise, resulting in improved discriminative power.
3. The improved accuracy and efficiency of our model has implications for various domains such as medical imaging, object recognition, and natural language processing, enabling more accurate and reliable classification in these applications.
4. The extensive experience is performed on challenging dataset and reveals that the proposed model achieved state-of-the-art performance when compared to existing techniques.

The rest of this manuscript is structured as follows: “Related work” section provides a concise summary of the existing techniques for lung nodule categorization reported in the literature. “Proposed model” section gives a detailed discussion of the materials and procedures used to treat pulmonary nodules. “Experimental results” section discusses the execution of the suggested model, as well as experimental data and the evaluation of the proposed model. Finally, in “Conclusion and future direction” section, we end the present work.

## Related work

Several investigations have employed deep learning methodologies to address classification issues^6–8^. The objective of this study is closely aligned with the existing computer-aided diagnosis (CAD) applications for the classification of lung nodules. Consequently, we conducted a thorough examination of the cutting-edge techniques for classifying lung nodules that have been recently developed.

Researchers have employed a two-dimensional convolutional neural network (CNN) to detect lung nodules in CT scans. This CNN focuses on extracting and learning important features from the two-dimensional images. For instance, in Ref.^9^, the authors developed a transfer learning technique using MultiResolution CNN to classify candidates in lung nodule recognition. They applied CNN-based image-wise calculations with different depth layers, resulting in improved accuracy of lung nodule detection. They achieved 0.9733 precision on the Luna 16 Data Set. In Ref.^10^, a CAD approach for pulmonary nodules was proposed, utilizing multi-view convolutional networks to reduce false positives. Another deep learning model, MultiView-KBC^11^, was proposed for lung nodule recognition. It employed KBC based deep learning technique to classify benign-malignant lung nodules on chest images. In Ref.^12^, a deep residual learning approach using CT scans was presented for cancer detection. ResNet and UNet models^13^ were employed for feature extraction, and machine learning algorithms (MLA) such as XGBoost and Random Forest were employed for classification, achieving 84% accuracy. They conducted a research study that used machine learning and ensemble learning methods to predict lung cancer based on early symptoms. They utilized various MLAs, including multilayer perceptron (MLP)^14^, Naïve Bayes, support vector machine (SVM)^15^, and neural networks for lung cancer classification. The dataset for this study was obtained from the UCI repository. The ensemble learning approach in this study achieved a 90% accuracy.

The 3D CNN, similar to its 2D counterpart, incorporates three dimensions in feature learning. It considers pairs of dimensions simultaneously, such as x and y, y and z, and z and x. To address false-positive reduction in lung nodule detection using chest radiographs, researchers^16^ developed an ensemble of CNNs. Another study^17^ introduced Multilevel contextual Encoding for false-positive reduction in chest radiographs, employing a fivefold cross-validation approach to detect nodule sizes and shapes. Their architecture achieved 87% sensitivity with an average of four false positives per scan. For detecting Small Cell Lung Cancer, a novel approach utilizing the entropy degradation method (EDM) was proposed. Researchers developed their own neural network (EDM) due to limitations in the dataset, using six healthy and six cancerous samples, achieving a detection accuracy of 77.8%. In another work^18^, machine learning techniques with image processing were employed for lung cancer detection. The data underwent various image processing techniques to enable the machine learning algorithm, and classification was done using a Support Vector Machine. In a different study^19^, Convolutional Neural Networks (CNN) combined with multiple pre-processing methods were explored. Deep learning played a significant role, demonstrating high accuracy and a low false-positive rate in automated labeling of scans.

The studies mentioned earlier do not employ an attention-based CNN deep learning model to identify lung nodules. Our objective is to utilize CNNs and customize their architecture to create a robust and effective tool for clinicians and researchers, enabling improved detection and classification of lung nodules. This, in turn, will contribute to better patient outcomes and enhanced healthcare. Previous research encountered challenges such as insufficient or small datasets for detection, resulting in limited subjects. These findings underscore the limited accuracy achieved with a higher number of machine learning or deep learning algorithms. Our proposed study intends to overcome these limitations and bridge the gaps in current research.

## Proposed model

**Figure 1:**
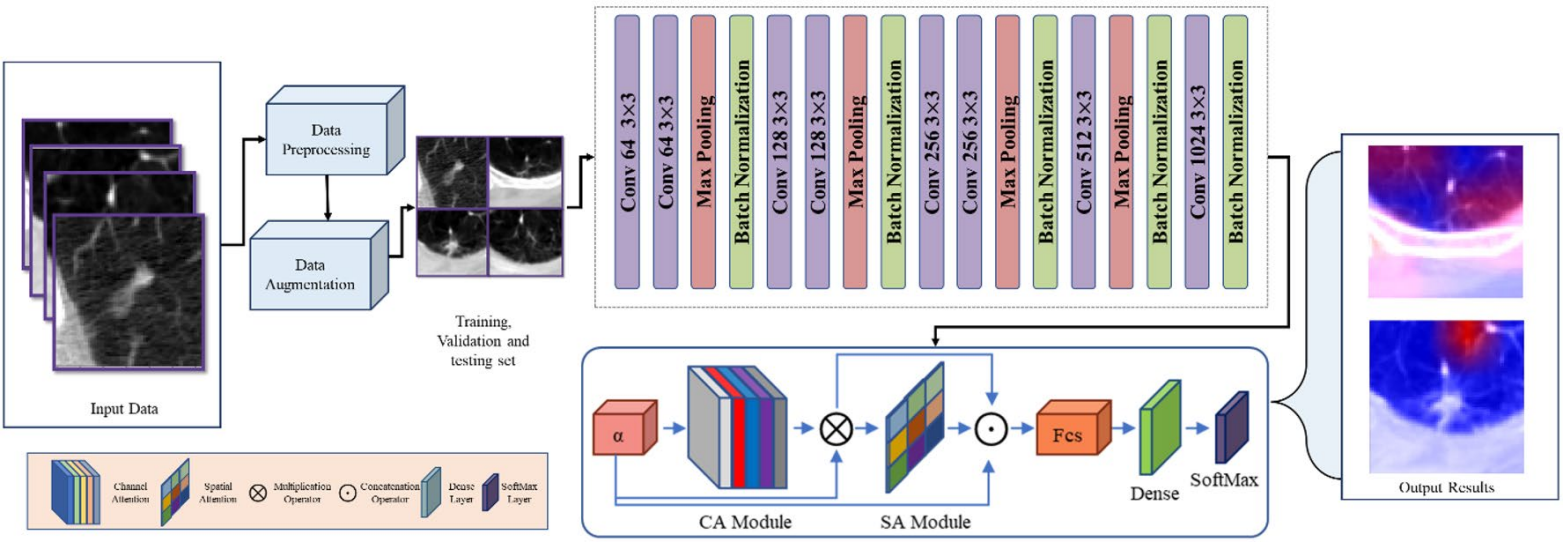
The overall flow of the proposed model for lunge nodule classification.

The proposed CNN-based attention model addresses two challenges in deep learning-based identification of malignant lung nodules: limited labeled samples and interference from complex background tissues. It leverages unlabeled CT scans to extract valuable image representations and learns fine-grained nodule features while defending against redundant information. The model comprises four components: data collection and preprocessing, data conversion and augmentation, deep feature extraction model, and a Dual Attention Module. The Dual Attention Module selectively focuses on relevant regions and features in the CT scans. The overall model structure is shown in Fig. 1, aiming to improve the accuracy of malignant lung nodule identification tasks by overcoming data limitations and background interference.

### Data collection and preprocessing

The process of data gathering is crucial in research as it directly influences the quality of the results. First, the leading attention is on combining a widespread dataset containing CT Scan images. This dataset is acquired from the LUNA 16 Data Set, which extensively aids in the successful achievement of research. Ensuring the collection of high-quality data is necessary to enable effortless interpretation by machines. All CT Scan images within the dataset are of uniform quality, facilitating clear presentation to medical professionals. The images in the LUNA Data Set are formatted as (.mhd) and (.raw) files, with the former containing header data and the latter containing multidimensional image data. To handle these images and initiate the preprocessing phase, the SimpleITK python library is utilized, allowing for the efficient reading of all .mhd files. The subsequent step in the proposed solution involves data preprocessing, which plays a critical role in transforming the data into a more comprehensible format for machines, enabling easier understanding and processing. This step is of utmost importance to ensure that the data is appropriately prepared to enhance machine comprehension. Within the LUNA 16 Data Set, the CT scans consist of n 512 × 512 axial scans, with each scan containing 200 images. Among these images, only 1351 are positive nodules, while the remainder are negative cases. Given the class imbalance, data augmentation techniques are employed to address this issue. Instead of training the CNN model on all original pixels, which would increase computational load and training time, a decision is made to crop the images based on the provided coordinates in the annotations. Figure 5 visually demonstrates an example of a cropped CT scan image from the dataset.

Additionally, it is noteworthy that all annotations within the LUNA 16 Data Set are initially represented in Cartesian coordinates and subsequently converted to voxel coordinates. Furthermore, the image intensity within the dataset is defined in the Hounsfield scale, requiring appropriate adjustment and rescaling for effective image processing. The images in the dataset are classified into two distinct categories: positive and negative. Nodule candidates marked as 1 are classified as positive, while those marked as 0 are classified as negative. Image labels are assigned accordingly to denote positive and negative instances. These labeled data are then utilized for training and testing purposes. Typically, the data is provided in the format of DICOM images or MHD/Raw files. Prior to feeding the data into any machine learning or deep learning model, it is crucial to convert the data into the required format, ensuring machines can effectively utilize and learn from it. Figure 5 provides a visual representation of the proposed system, further illustrating its components and functionalities.

### Data conversion and augmentation

The subsequent stage involves converting all the pre-processed data into the JPEG format to ensure computer comprehensibility. JPEG format is easily readable by both humans and computers, allowing for visual verification of the desired format. This format enables convenient viewing and assessment of the images. Additionally, the data is transformed into smaller 50 × 50 images to reduce data size and minimize computational power requirements. Larger data sizes tend to consume substantial computing resources, and downsizing the images to 50 × 50 dimensions helps address this issue. In cases where there is an imbalance in the data, it is crucial to augment the dataset. Since the data was not balanced, manual data augmentation techniques are employed. Data augmentation^20^ plays a significant role in addressing the class imbalance by rotating the images in various directions and creating additional copies from different angles. This approach generates more diverse instances of the same data, effectively mitigating the data imbalance problem. To facilitate image pre-processing and data augmentation, the Keras Image Data Generator is utilized. Keras Image Augmentation techniques encompass zooming in and out, exploring different image data shear ranges, and flipping the images. These essential steps ensure that the data is processed in multiple ways, allowing the machines to learn and interpret the data from various perspectives.

### Proposed network architecture

In our study, we have developed a custom CNN architecture that incorporates channel and spatial attention mechanisms, setting it apart from other CNN models. The sequential structure of our model consists of multiple convolutional layers, which effectively extract informative features from the input images through the application of filters. Furthermore, the inclusion of max pooling layers helps in reducing the spatial dimensions of the feature maps, while batch normalization layers ensure improved stability and efficiency of the model as shown in Fig. 1.

#### Deep features extraction model

**Figure 2:**
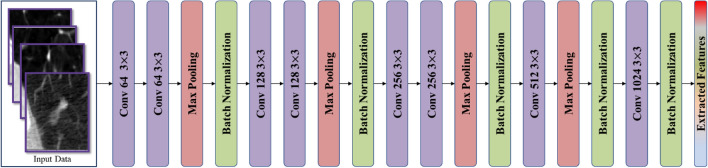
The structure of proposed modified CNN model.

CNNs have demonstrated exceptional capabilities in extracting valuable features as of raw images, making them highly appropriate for a wide range of computer vision applications^21^. However, selecting the most appropriate CNN architecture for a specific domain can be a complex endeavor. In order to ensure accurate forecasts in real-world circumstances, a balance must be struck between computing complexity and assurance. There has been a lot of study done in the field of vision-based medical picture analysis. Researchers frequently use pre-trained paradigms as the basis for feature extraction and then fine-tune them on object datasets to obtain accurate localisation and categorization. By using the learned weights and parameters from pre-trained paradigms, fine-tuning allows the system to acquire spatial data efficiently. Because pre-trained networks offer a robust and diverse feature extraction pipeline, they are highly advantageous for initializing networks in vision-based recognition tasks. We have created a modified CNN architecture specifically made for lung nodule classification tasks, even in extremely difficult settings. This architecture was inspired by the feeling of current feature extraction algorithms in many computer vision domains. Our architecture comprises specific layers and modules that improve its capacity to precisely categorize these nodules while taking into account the particular characteristics of lung nodules. To analyze lung nodule images efficiently, a comprehensive set of layers is incorporated into the proposed architecture. A matching input tensor is initialized, and the input layer is configured with a shape of (64, 64, 1). The architecture features numerous convolutional blocks, every single compiled of Conv2D layers with variable filter sizes, kernel sizes, ReLU activations, and uniform padding. MaxPooling2D layers with suitable pooling sizes are utilized for down-sampling, followed by BatchNormalization layers for regularization. The network combines a Bottleneck Attention Module (BAM) to selectively emphasize fundamental features, improving the model’s discriminative capabilities as demonstrated in Fig. 2. Afterwards, global average pooling is operated to summarize spatial information. The fully connected layers comprise a Dense layer with 512 units and ReLU activation, resulted by a Dropout layer with a dropout rate of 0.5 to avoid overfitting. The output layer contains of a Dense layer with 2 units for binary classification using softmax activation. We have improved the effectiveness and dependability of lung nodule categorization systems, which will lead to more accurate diagnosis and treatment of lung-related illnesses. To accurately extract features from lung nodule pictures, our customized CNN architecture employs cutting-edge techniques including convolutional layers, pooling layers, and fully linked layers. We also use cutting-edge approaches such as transfer learning and attention processes to improve the network’s capacity to discriminate between different types of nodules. The attention mechanisms assist the network in recognizing subtle patterns and features required for effective classification by focusing on the most important portions of the pictures. Furthermore, our architecture considers the difficulties presented by exceedingly complicated scenarios. Variations in nodule size, shape, and appearance are among these situations, as are low-resolution pictures and noisy data. To overcome these issues, we employ multi-scale processing, data augmentation, and robust regularization algorithms to increase the network’s generalization and resilience. We proved the efficiency of our customized CNN architecture in reliably identifying lung nodules after thorough testing and analysis on different datasets. Our design beats previous methodologies, indicating that it has the potential to dramatically enhance research into lung nodule analysis and diagnosis. We aim to provide researchers and clinicians with a dependable and efficient tool for improving the identification and classification of lung nodules by utilizing CNNs and customizing the architecture to the particular task at hand. This will ultimately improve patient outcomes and healthcare.

#### Dual attention module

**Figure 3:**
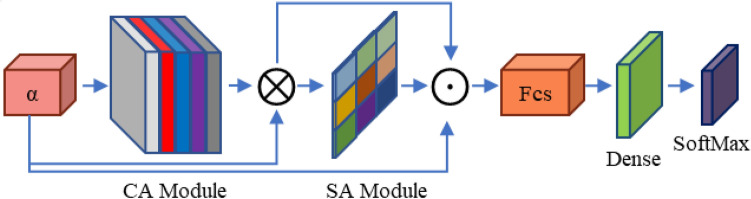
The structure of proposed dual attention mechanism.

Several domains of application have explored the combination of convolutional neural network (CNN) architectures and attention processes^22–25^. These architectures have proved significant promise when processing video data, where the similarity between the frames in a series is repeatedly relatively strong. However, due to the variety of the data and the employ of distinct channel attention (CA) or spatial attention (SA) components, attention-based techniques have participated in hurdles when dealing with image data. For image-based detection, earlier works have tried to build simply a CA module into CNN architectures^26^. Whereas the usage of a CA component with backbone-model features has been functioning for recognizing uncomplicated objects in simpler circumstances, such as those, its performance is motionless constrained when dealing with more complex conditions, particularly in fields like medical imaging. In this study, we design a brand-new model that joins both the CA and SA modules. We call this model a “lung nodule attention portion”. Employing both of these attention methods, our model is able to focus on the areas that are most important for precise nodule classification. Through its dual attention strategy, the dual attention module we introduced successfully crops crucial lung nodule regions and achieves accurate localization. Our model can effectively capture meaningful channel-wise correlations in the image information by integrating the CA module. As a result, it can recognize important patterns and characteristics that help classify lung nodules. Furthermore, the SA module increases the model’s capability to pay consideration to spatial features, enabling it to identify specific sites of interest within the lung nodules. Our proposed technique provides a detailed method of lung nodule classification by containing the uses of both attention mechanisms as illustrated in Fig. 3. It leverages the power of channel-wise correlations and spatial information to ensure accurate localization and valuable feature extraction. We anticipate that this dual attention scheme will significantly enhance the performance of our model in complex scenarios, particularly in the field of medical imaging where precise identification and classification of lung nodules are critical for early detection and diagnosis.

1. *Channel attention mechanism* The human visual attention system, which simulates how humans focus on various objects, serves as an inspiration for the attention mechanism in this situation. The CA component aims to identify the most significant “what” in an input image. When it comes to information aggregation, actual data from CBAM shows that integrating both average- and max-pooled features strengthens the network’s representation capacity substantially more than performing each process separately. The process begins by aggregating global information from a feature map through average pooling and max-pooling operations. This generates two distinct descriptors: ${\chi }_{AUG}^{\mathcal{C}}$ and ${\chi }_{Max}^{\mathcal{C}}$. These descriptors then pass through a scale network, which produces a channel attention map (MLP) denoted as ${\mathcal{M}}_{\mathcal{C}}\in {\mathcal{R}}^{\mathcal{C}/2 \mathcal{G}\times 1\times 1}$ . The channel attention map is used to adjust the shape of $\chi$, enabling subsequent element-wise summation with the sub-feature. Next, average pooling and max-pooling operations are applied to both branches of each sub-feature $\chi {\mathbb{K}}$. The output feature vectors are then merged using element-wise summation, resulting in ${\chi }_{\mathcal{C}}\in {\mathcal{R}}^{\mathcal{C}/2 \mathcal{G}\times 1\times 1}$. This computation can be represented as the following equation:
  1 $${\mathcal{M}}_{\mathcal{C}} \left(\chi \right)=MLP\left(AVG(\chi )\right)+MLP\left(MAXPool(\chi )\right),$$
  2 $$\chi =MLP\left( {\chi }_{{\mathbb{K}}1}+{\chi }_{{\mathbb{K}}2})\right)+MAXPool\left({\chi }_{{\mathbb{K}}1}+{\chi }_{{\mathbb{K}}2}\right)+{\mathcal{M}}_{\mathcal{C}} \left(\chi \right).$$
2. In addition to the channel attention mechanism, a compact feature is constructed to facilitate accurate and adaptable selection assistance. This is accomplished through a simple gating mechanism with sigmoid activation. The final output of the channel attention is obtained as follows:
  3 $$\chi \mathcal{C}=\delta \left(FC (\chi )\right)\cdot {\chi }_{{\mathbb{K}}1}=\delta \left({\mathbb{W}}_{\mathcal{C}b}+bc\right)\cdot {\chi }_{{\mathbb{K}}1}.$$
3. *Spatial attention mechanism* Spatial attention and channel attention serve different purposes in feature maps. While channel attention focuses on determining the importance of “what” features within the feature map, spatial attention is concerned with identifying “where” the key feature information is located. We employ Group Normalization (GN) over the ${\chi }_{{\mathbb{K}}1}$ and ${\chi }_{{\mathbb{K}}2}$ branches in order to calculate spatial attention. This makes it easier on the computer while guaranteeing that spatial information about the item is used properly, giving the feature extraction network more precise data. The computation of spatial attention is represented as follows:
  4 $$\chi \mathcal{S}=\delta \left({\mathbb{W}}\mathcal{S}\cdot \left(GN\left({\chi }_{{\mathbb{K}}2}\right)+GN\left({\chi }_{{\mathbb{K}}1}\right)\right)+bs\right)\cdot {\chi }_{{\mathbb{K}}2}.$$
  Here, ${\mathbb{W}}\mathcal{S}$ and bs are parameters with a shape of ${\mathcal{R}}^{{{\mathcal{C}}/2~~{\mathcal{G}} \times 1 \times 1}}$. The two branches, ${\chi }_{{\mathbb{K}}1}$ and ${\chi }_{{\mathbb{K}}2}$, are then merged together to match the number of channels to the number of inputs. This integration facilitates the utilization of spatial attention to enhance the feature map representation.

Our work significantly contributes to the field of image classification by introducing a custom CNN architecture with integrated channel and spatial attention mechanisms. Attention mechanisms that selectively focus on pertinent characteristics while ignoring noise and unimportant information are introduced to improve the feature extraction process. This improves the image categorization accuracy significantly. By simplifying the learning of attention weights for each channel, the channel attention technique allows the model to prioritize informative channels while suppressing less important ones. It consequently detects important patterns and distinctive features in the data. The spatial connections and dependencies between the features are also captured by the spatial attention mechanism. By concentrating on pertinent geographical regions, the model is able to identify and extract meaningful spatial patterns that enhance classification performance. Compared to conventional CNN architectures, our model’s incorporation of these attention mechanisms enhances accuracy and discriminative power by enabling us to identify and emphasize the most important characteristics. Our work provides a substantial improvement in image classification by exploiting attention mechanisms and resolving the drawbacks of conventional CNNs. This research will be beneficial to many domains, such as medical imaging, object recognition, and natural language processing, since it creates new avenues for the construction of more precise and effective classification models. In medical image analysis, attention processes are particularly important for lung nodule detection. These processes, which comprise CA and SA, play a major role in enhancing the accuracy of precisely localizing abnormalities and recognizing subtle patterns in medical imaging. Conventional convolutional neural networks face difficulties in recognizing lung nodules due to the intricate details included in medical imaging. Models can focus on the most salient features (“what”) in the image because CA is based on the human visual attention system. This is crucial in order to distinguish relevant nodule features from the rest of the picture data. But because SA focuses on “where” important feature information is situated, it is extremely useful for pinpointing specific regions of interest within the lung nodules. For precise and accurate localization, especially when it comes to lung nodule classification, this spatial awareness is crucial. The authors’ model successfully captures the essential channel-wise correlations and spatial details needed for precise lung nodule detection by combining CA and SA in their “lung nodule attention module.” The model performs much better thanks to this dual attention approach, particularly in the difficult and important field of medical imaging, where accurate lung nodule identification is essential for early diagnosis and detection.

## Experimental results

### Model implementation

The proposed model was assessed using the publicly available LUNA 16 dataset, which was split into a training set and a testing set, each containing 80% of the data. To prepare the CT scans for input into the network, they were split into three multi-scale patches of different sizes: 64 × 64 × 64, 32 × 32 × 32, and 16 × 16 × 16. These patches were fed into the network, enabling the model to collect data at various sizes. During training, several hyperparameters were determined. The learning rate was first set at 0.01 and the utmost number of epochs was controlled to 150. A weight decay of 1 × 10^−4^ was employed to avoid overfitting. As the training proceeded, specific checkpoints changed the learning rate. After 50% of the epochs, it decreased to 0.001, then 0.001 after 75% of the epochs, and finally 0.0001 after 90% of the epochs. These adjustments enhanced the training procedure. A stochastic gradient descent optimizer with a momentum of 0.9 was utilized to improve the performance of the model. During the model’s training process, the binary cross-entropy loss function was utilized, which was vital. This specific loss function is appropriate for binary classification tasks, where pixels are categorized as either belonging to a target region or not, such as the segmentation of medical pictures. Binary cross entropy penalizes pixel-by-pixel departures from the ground truth, assisting the model in spotting intricate patterns in the data. In the end, this produces segmentation results that are more accurate. The adaptive adjustments in learning rate during training further refined the model’s performance, resulting in improved generalization and effective convergence throughout the dataset. In order to minimize the difference between the real ground truth data and the model outputs, this optimizer was crucial. Through iterative adjustments to the model’s parameters based on the computed gradients, the optimizer facilitated the model’s convergence towards a more precise representation of the underlying patterns in the data.

### Model evaluation metrics

This study employed a number of widely accepted criteria to assess how well the proposed approach performed in classifying nodules^15,27^. These metrics assess the model’s ability to differentiate between benign and malignant lung nodules. The following are the evaluation metrics for the proposed model.

Sensitivity or recall is defined as the ratio of correctly diagnosed malignant nodules to the total number of malignant nodules. This figure sheds light on the model’s ability to consistently identify malignant cases, which is essential for accurate sickness detection. The overall correct classification rate is represented by accuracy, which is a crucial metric. It provides a general idea of the model’s ability to identify benign and malignant nodules. The percentage of malignant nodules that are correctly identified among all nodules that are predicted to be malignant is known as precision. This figure illustrates how well the model can detect malignant conditions while reducing false positives. Specificity is the percentage of benign nodules that are accurately identified. This metric highlights how successfully the model classified cases as benign, which adds to a comprehensive assessment of its effectiveness. AUC is a widely used metric in the assessment of binary classification techniques. Area Under the Receiver Operating Characteristic Curve is abbreviated as AUC. It assesses how well the model can differentiate between benign and malignant nodules while accounting for differences in the dataset. The F1-score offers a fair evaluation of the model’s capacity to distinguish between benign and malignant nodules. It is computed as the harmonic average of precision and recall. It offers a thorough perspective that balances accuracy and sensitivity. These measures, taken together, contribute to a detailed investigation of the proposed model’s performance in lung nodule categorization. The following evaluation metrics can be computed:

5 $$Accuracy={T}^{+}+\frac{{T}^{-}}{{T}^{+}}+{F}^{+}+ {T}^{-}+{F}^{-},$$

6 $$Sensitivity ={ T}^{+}/{T}^{+}+{F}^{-},$$

7 $$Specificity ={T}^{-}/ {T}^{-}+{F}^{+},$$

8 $$Precision ={ T}^{+}/{T}^{+}+{F}^{+},$$

9 $$F1-score ={2 \times T}^{+}/ \left({2 \times T}^{+}+{F}^{+}+{F}^{-}\right).$$

The terms ${{\text{T}}}^{+}$, ${{\text{T}}}^{-}$, ${{\text{F}}}^{-}$, and ${{\text{F}}}^{+}$ are used in the following equations. True positives are nodules that have been appropriately recognized as benign and cancerous. ${{\text{T}}}^{-}$ stands for true negativity and counts the number of correctly identified benign nodules. False negative, or ${{\text{F}}}^{-}$, denotes the number of cancerous nodules that were wrongly identified as benign. False positive, or ${{\text{F}}}^{+}$, refers to the number of benign nodules that were mistakenly labeled as cancer.

### Discussion

**Figure 4:**
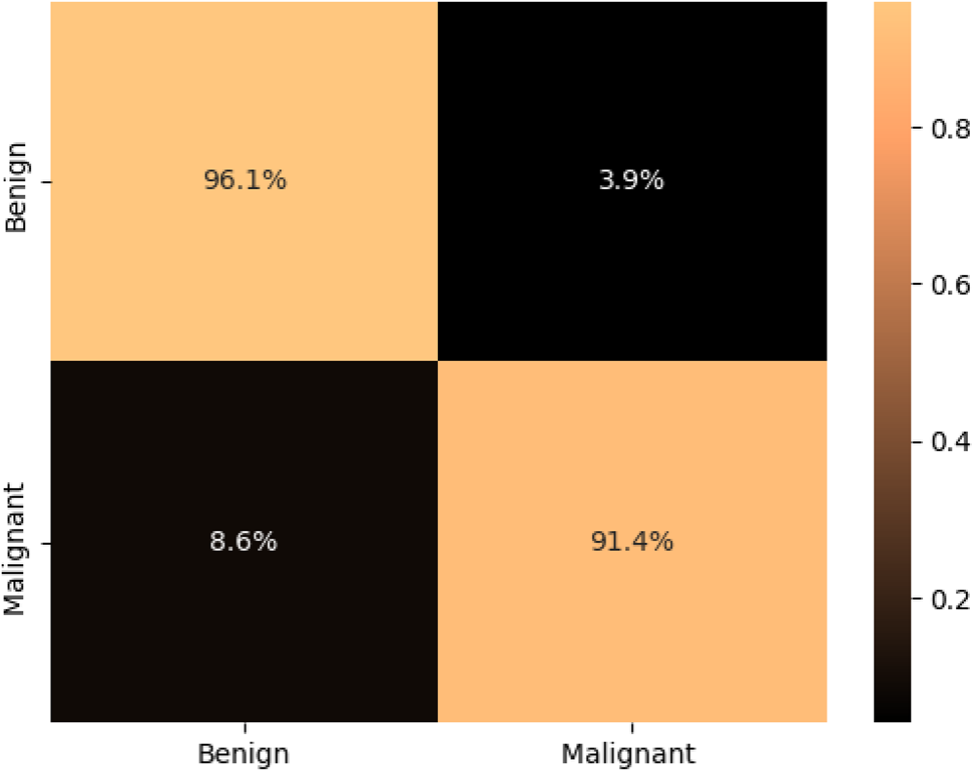
The confusion matrix of the proposed model using Luna16 dataset.

**Figure 5:**
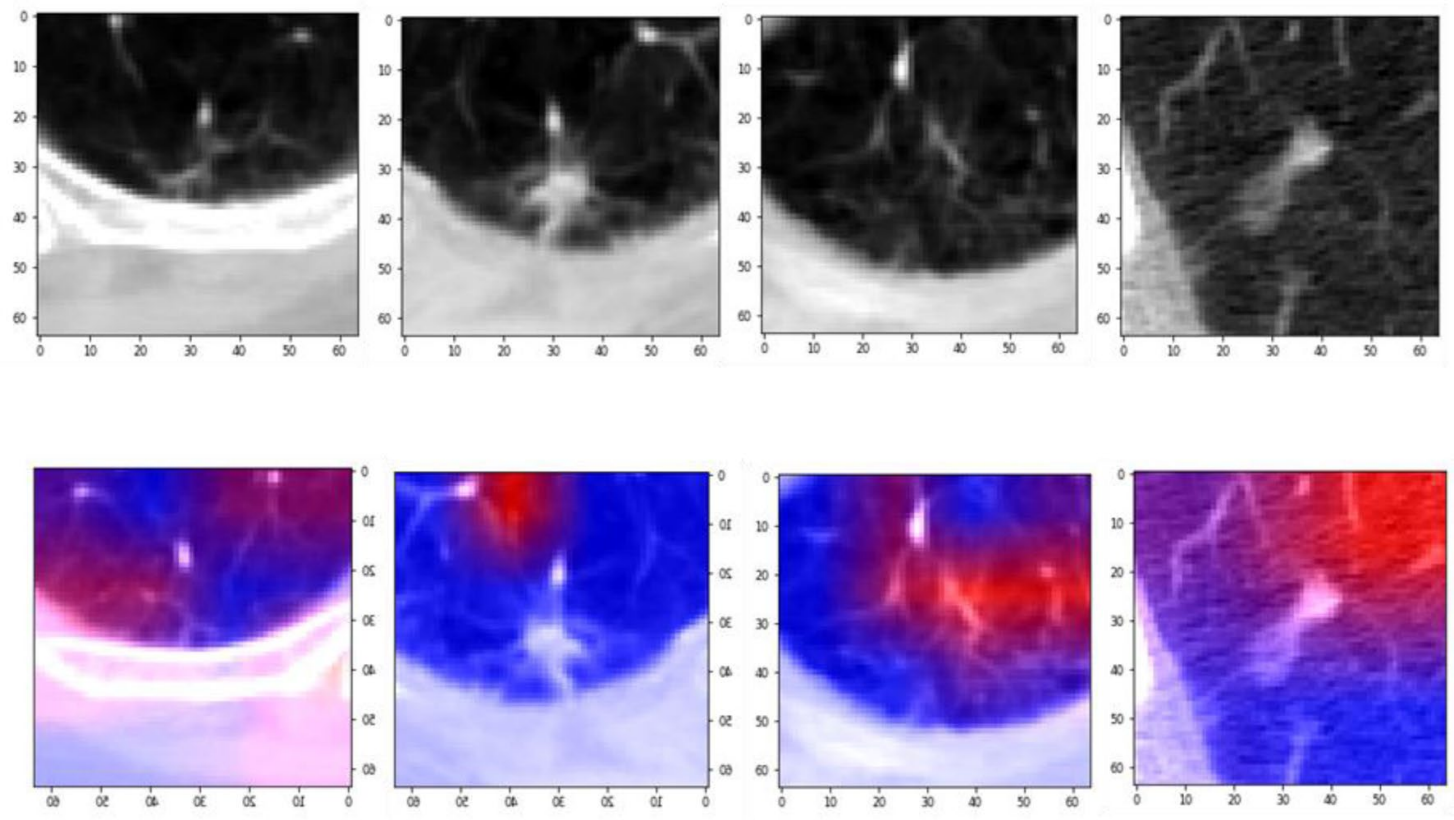
Visual analysis of the proposed model using LUNA 16 dataset.

**Figure 6:**
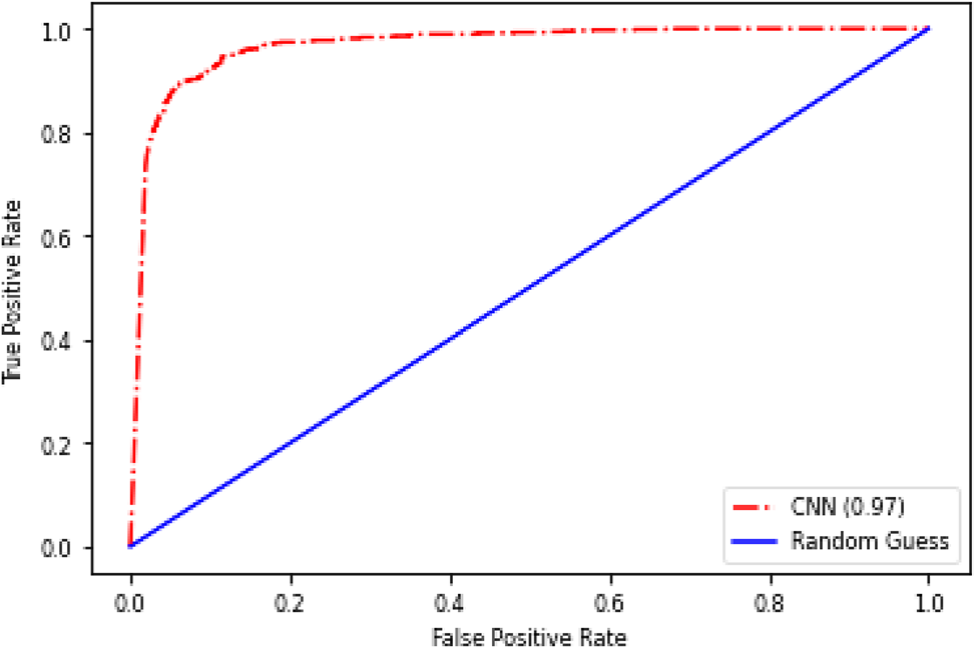
The RoC of the proposed model using LUNA16 dataset.

In Table 1, an in-depth analysis is presented, evaluating various lung nodule detection systems that underwent validation using the LUNA16 public dataset. This comparison allows for a comprehensive understanding of the proposed method’s performance. The majority of the methods examined in Table 1 employed deep learning techniques, which leverage automated feature extraction to achieve accurate lung nodule detection. These deep learning-based approaches showcased higher detection sensitivities and demonstrated superior network generalization in comparison to machine learning approaches. Among the methods analyzed, ST^28^, TSDID^29^, Multi-taskCNN^30^, TL-basedCNN^31^, and KBC^11^ utilized 2D patches as input for their networks. However, this approach overlooked the spatial contextual features inherent in the three-dimensional (3D) nature of CT images. Consequently, the reliance on 2D-based architectures resulted in relatively lower detection sensitivities when compared to their 3D-based counterparts. On the other hand, the 3D-based models discussed in Table 1 effectively extracted spatial features that were conducive to accurate nodule candidate detection. However, it is important to note that these 3D models typically involved a higher number of parameters, leading to increased memory storage requirements. When applying such models in real-world circumstances, this factor must be taken into account. When choosing a good detection system, it is essential to balance the need for accuracy with available processing power. In addition to the analysis in Table 1, the preceding figures provide further insights. The confusion matrix delves into the suggested model’s true positive, true negative, false positive, and false negative predictions. This matrix makes it easier to evaluate the model’s performance and identify potential flaws. The trade-off between the true positive rate and the false positive rate at various categorization levels is seen in Fig. 6’s receiver operating characteristic (ROC) curve. A thorough analysis of the model’s effectiveness over numerous choice boundaries is given by this visual depiction. Moreover, the suggested model’s real output is glimpsed through graphic representations like Figs. 4 and 6. The confusion matrix of the proposed model is shown in Fig. 4, visual analysis of the proposed model is presented in Fig. 5, and the ROC curve of the proposed model is shown in Fig. 6. We design a 2D attention mechanism that performs better in terms of F1-score (95.24%), AUC (98.00%), specificity (93.17%), accuracy (94.40%), precision (95.80%), and sensitivity (94.69%). These metrics demonstrate the model’s ability to discriminate between favorable and unfavorable scenarios, retain high accuracy, consistently identify favorable cases, and strike a balance between sensitivity and precision. Finally, our 2D attention-based model performs better than other assessment measures, suggesting that it might be used as a lung nodule classification method. By using attention processes, it strikes a balance between processing economy and accuracy, which is essential for real-world applications in the field of medical imaging. Using deep learning techniques to produce high detection sensitivities and superior network generalization while effectively capturing 3D spatial data in CT images is one of our approach’s strengths. In addition, the integration of visual representations provides useful insights into the model’s decision-making process. On the contrary, 3D-based models, including ours, have a greater number of parameters, which increases memory storage requirements and may be a constraint in resource-constrained contexts. Furthermore, the computational demands of 3D models necessitate a balance between accuracy and available computing capacity. Finally, in real implementations, the trade-offs in decision thresholds, as represented by the ROC curve, must be carefully evaluated.

**Table 1:** Performance comparison of the proposed model with state-of-the-arts.

Methods	2D/3D	Sensitivity	Precision	Accuracy	AUC	Specificity	F1-score
ST^28^	2D	89.73	–	–	95.05	86.36	–
TSDID^29^		84.19	–	89.53	96.65	92.02	–
Multi-taskCNN^30^		87.74	–	–	95.59	88.87	–
TL-basedCNN^31^		85.38	73.48	88.41	93.19	–	78.83
KBC^11^		86.52	87.75	91.60	95.70	94.00	87.13
Multi EnsemModel^39^		60.26	–	86.79	–	95.42	–
DC-GAN^32^		89.35	–	92.07	92.08	94.8	–
DLG^9^		88.66	87.38	88.46	95.62	–	–
MTL^40^	3D –	–	–	91.26	–	–	–
BTNet^41^		–	–	88.31	93.15	–	–
Deep3DDPN^42^		–	–	90.44	–	–	–
SS-OLHF-^33^		82.60	–	88.66	93.03	91.95	–
Multi-cropCNN^34^		77	–	87.14	93	93	–
MMEL-3DCNN^35^		83.7	–	90.6	93.9	93.9	–
PFSC^36^		84.45	92.07	84.26	91.6	83.84	88.01
SSTL-DA^37^		90.93	91.18	91.07	95.84	91.22	91.00
SE-ResNeXt^43^		–	–	91.67	95.63	–	–
SEN-MSAN^44^		91.3	–	91.9	–	–	91
STLF-VA		91.62	92.99	92.36	97.17	93.08	92.25
MVCN^10^		–	–	94	–	–	
EnsemCNN^16^		–	–	87	–	–	
NBA^12^		–	–	84	–	–	
EMLC^45^		–	–	90	–	–	
Ensem2DCNN^38^		–	–	95	–	–	
Proposed model	2D_attention	94.69	95.80	95.40	98.00	93.17	95.24

### Comparison with state-of the art

The proposed model, which is a 2D attention-based model, achieves superior performance compared to state-of-the-art methods in various evaluation metrics, as shown in Table 1. In terms of sensitivity, the proposed model achieves a sensitivity of 94.69%, indicating its ability to accurately detect positive cases. This outperforms methods such as ST^28^, TSDID^29^, Multi-taskCNN^30^, Multi-taskCNN^30^, KBC^11^, DC-GAN^32^, DLG^9^, SS-OLHF-^33^, Multi-cropCNN^34^, MMEL-3DCNN^35^, PFSC^36^, and SSTL-DA^37^. For precision, the proposed model reaches an accuracy of 95.80%, revealing a high level of accuracy in correctly identifying positive cases. It outperforms most methods except for DLG^9^, KBC^11^, PFSC^36^, SSTL-DA^37^, and Ensem2DCNN^38^. In terms of accuracy, the proposed model achieves an accuracy of 94.40%, surpassing methods like ST^28^, TSDID^29^, Multi-taskCNN^30^, TL-basedCNN^31^, SS-OLHF-^33^, Multi-cropCNN^34^, and MMEL-3DCNN^35^. The proposed approach also outperforms in terms of AUC, with an AUC score of 98.00%. This demonstrates its capacity to correctly discern between positive and negative situations, exceeding all other strategies in the table. When specificity is taken into account, the suggested model achieves a specificity of 93.17%, proving its ability to reliably detect negative situations. It outperforms techniques such as ST^28^, TSDID^29^, Multi-taskCNN^30^, and TL-basedCNN^31^. Finally, in terms of F1-score, the proposed model obtains a score of 95.24%, showing a fair compromise between accuracy and sensitivity. It outperforms most techniques except for DLG^9^, KBC^11^, PFSC^36^, and SSTL-DA^37^. The proposed 2D attention-based model performs better overall on a wide range of assessment measures, which indicates that it is a viable method for the particular classification problem.

### Detailed ablation study

In the section that follows, the efficacy of several solo baseline-CNNs and CNNs boosted with attention mechanisms was carefully examined using the Luna 16 dataset. The primary objective of the evaluation was to determine their capacity to identify complex patterns in medical photographs, particularly those pertaining to lung nodules. The results for each approach are shown in Table 2, both with and without the attention mechanisms included. Strong evidence of the recommended model’s superiority in both testing scenarios was found by our research. When attention mechanisms were not included in the first analysis, the models produced poor performance, highlighting the importance of attention mechanisms in improving model performance. In contrast, our proposed model constantly outperformed its competitors, revealing its essential ability to recognize difficult patterns without the use of attention mechanisms. In the subsequent test, attention mechanisms were integrated into each model to evaluate their impact on overall performance. Notably, the results indicated a general enhancement in the performance of all models, highlighting the efficacy of attention mechanisms in refining the models’ ability to discern relevant features. However, what stands out is the consistent dominance of the proposed model, which not only exhibited improvement but surpassed existing methods by a substantial margin. This not only validates the effectiveness of attention mechanisms but also underscores the robustness and adaptability of our proposed model to leverage these mechanisms for increased performance. Thus, in both tests, our proposed model excelled over other established approaches, as evidenced in Table 2. This underscores its efficacy in detecting complex lung nodule abnormalities, especially in low-resolution images, making it well-suited for real-time medical image analysis.

**Table 2:** The detailed ablation studies of various backbone CNN models with attention and without attention mechanism.

Dataset	Model	Attention mechanism	Sensitivity	Precision	Accuracy	Specificity	F1-score
Luna 16	EfficientNetB0	✓	93.50	95.60	95.30	93.00	95.00
		×	92.40	95.50	95.20	93.10	94.90
	EfficientNetB1	✓	94.40	95.70	95.20	93.10	94.90
		×	94.30	95.60	95.10	93.20	94.80
	EfficientNetB2	✓	94.60	95.50	95.40	93.20	95.10
		×	94.70	95.40	95.50	93.30	94.80
	EfficientNetB3	✓	94.60	95.30	95.40	93.40	94.70
		×	94.55	95.65	95.35	93.05	94.95
	EfficientNetV2B0	✓	94.45	95.55	95.25	93.15	94.85
		×	94.45	95.75	95.25	93.15	94.85
	EfficientNetV2B1	✓	94.35	95.65	95.15	93.25	94.75
		×	94.65	95.55	95.45	93.25	95.05
	EfficientNetV2B2	✓	94.55	95.45	95.35	93.35	94.95
		×	94.75	95.35	95.55	93.35	94.75
	NASNetMobile	✓	86.65	88.25	87.45	86.45	87.65
		×	86.69	88.80	87.40	86.17	86.24
	DenseNet121	✓	89.10	86.80	88.50	90.20	87.50
		×	89.20	86.90	88.60	90.30	87.60
	MobileNetV2	✓	88.80	87.30	89.00	88.10	87.90
		×	88.90	87.20	89.10	88.20	87.80
	MobileNet	✓	88.50	87.90	88.80	88.40	87.40
		×	88.60	87.80	88.90	88.50	87.30

## Conclusion and future direction

Cancer remains a significant public health issue with a high mortality rate, and despite the billions of dollars invested in research, the disease still poses unanswered questions. Cancer research is a continuous process that requires ongoing efforts since no definitive solutions have been developed to date. There is currently no universally accepted standard for cancer detection and prediction, making cancer research an ongoing open question that deserves more attention. Recent research studies on cancer using current datasets provide valuable insights and statistics on the progress made thus far in detecting and predicting cancer. Such research may shed light on the latest causes and warning signs of cancer, providing a foundation for further research and innovation in the field. Our study introduces a novel convolutional neural network (CNN) architecture tailored for the accurate analysis of lung nodule images. Through the incorporation of a dual attention mechanism, our custom CNN effectively identifies and emphasizes the most informative features within the images. The attention module combines both channel attention and spatial attention mechanisms to selectively highlight the crucial features, enhancing the model’s performance. Additionally, global average pooling is utilized to summarize spatial information. The performance of the model was evaluated by extensive trials on the lung nodule benchmark dataset. The study’s findings demonstrate the enhanced efficacy of our suggested model, outperforming other models in terms of accuracy and setting a new benchmark for lung nodule detection and classification tasks. The results of our study have important ramifications. We support current efforts to enhance early detection and diagnosis of lung cancer by developing a more precise and effective model for lung nodule analysis. This might enable prompt intervention and treatment, perhaps saving lives. Our dual attention method further highlights the significance of using sophisticated attention mechanisms in medical picture processing, which can have wider applications in many healthcare domains. Overall, our study lays the groundwork for future advances in cancer detection and medical image processing, opening the path for novel solutions to this important global health issue.

## Data Availability

The datasets used and/or analyzed during the current study are available from the corresponding author upon reasonable request.
